# OMRT-14. Small molecule circadian clock compounds exhibit potential as a novel therapy paradigm for glioblastoma

**DOI:** 10.1093/noajnl/vdab070.038

**Published:** 2021-07-05

**Authors:** Priscilla Chan, Tsuyoshi Hirota, Meng Qu, Guoxin Zhang, Khalid Shah, Hiroaki Wakimoto, Theodore Kamenecka, Laura Solt, Jeremy Rich, Steve Kay

**Affiliations:** 1 University of Southern California, Los Angeles, CA, USA; 2 Nagoya University, Furocho, Chikusa Ward, Nagoya, Japan; 3 University of California, San Diego, La Jolla, CA, USA; 4 Harvard Medical School, Boston, MA, USA; 5 Massachusetts General Hospital Cancer Center, Boston, MA, USA; 6 Scripps Florida, The Scripps Research Institute, Jupiter, FL, USA; 7 University of Pittsburgh, Pittsburgh, PA, USA

## Abstract

Glioblastoma multiforme (GBM) is the most prevalent and aggressive primary brain tumor type, claiming the lives of patients within 2 years of diagnosis. The major challenges in treating GBM are largely due to the biological characteristics of the tumor and the brain and pharmacokinetics of many drugs approved for other cancers. These tumors are located in areas that make it difficult to surgically resect without posing major issues and exposure to many drugs and therapies are limited due to the blood-brain barrier (BBB). GBMs also contain cancer stem cells, called GSCs, that have self-renewal and tumor initiating abilities, can secrete angiogenic factors, invade into the normal brain, and are chemoresistant and radioresistant. We found that GSCs have an exclusive dependence on core circadian clock transcription factors, Brain and Muscle ARNT-Like 1 (BMAL1) and Circadian Locomotor Output Cycles Kaput (CLOCK). These results suggest the potential for small molecule modulators of the circadian clock as a novel therapy paradigm for GBM treatment following surgical resection to prevent GSC infiltration and reoccurrence of the primary tumor. Here we found that multiple classes of clock compounds (Cryptochrome (CRY) stabilizers, REV-ERB agonists, Casein Kinase 1 (CK1) inhibitors, and Casein Kinase 2 (CK2) inhibitors) have the ability to elongate circadian periods in a clock reporter cell line. They also selectively and potently target patient-derived GSCs that range in sensitivity to temozolomide (TMZ) chemotherapy treatment while having limited effects on control cells both as single agents and in combination with each other. This data provides a platform for further exploration of synergistic effects of combining clock compounds with each other or with current GBM therapies, such as chemotherapy and radiation, with the ultimate goal of developing a clinical model of treatment.

